# Creutzfeldt–Jakob disease‐like imaging in valosin‐containing protein D395G mutation

**DOI:** 10.1002/alz.70816

**Published:** 2025-10-16

**Authors:** Ryota Kobayashi, Masafumi Kanoto, Shinobu Kawakatsu, Akihito Suzuki

**Affiliations:** ^1^ Department of Psychiatry Yamagata University School of Medicine Yamagata Japan; ^2^ Department of Diagnostic Radiology Yamagata University School of Medicine Yamagata Japan; ^3^ Department of Neuropsychiatry Aizu Medical Center Fukushima Medical University Aizuwakamatsu Japan

1

Dear Editor:

We read the recent study by Watanabe et al.^1^ on vacuolar tauopathy associated with a *D395G* mutation in the valosin‐containing protein (VCP), published in this journal, with great interest. Based on five cases with the VCP *D395G* mutation, the authors reported that a ribbon‐like hyperintensity in the occipital cortex on diffusion‐weighted magnetic resonance imaging (DWI) may be a characteristic neuroradiological finding associated with this mutation.^1^ Their analysis of three autopsy cases revealed that DWI hyperintensity typically corresponded to vacuolar changes in brain tissue.^1^

We previously reported the clinical course and pathological findings of a Japanese individual with the VCP *D395G* mutation.^2^, ^3^ Motivated by the findings of Watanabe et al.,^1^ we retrospectively reviewed the longitudinal changes in fluid‐attenuated inversion recovery (FLAIR) imaging and DWI in our case. The patient, who presented with typical behavioral variant frontotemporal dementia symptoms, showed no signal abnormalities in the occipital cortex on either FLAIR imaging or DWI at the initial visit (Figure 1A). However, a magnetic resonance imaging (MRI) scan conducted 3 years after the initial visit showed a ribbon‐like hyperintensity in the occipital cortex on DWI, with no apparent changes on FLAIR imaging (Figure 1B). A subsequent MRI scan conducted 10 years after the initial visit revealed slight hyperintensity in the occipital cortex on FLAIR imaging, in addition to a persistent ribbon‐like hyperintensity on DWI (Figure 1C). The areas of hyperintensity on FLAIR imaging and DWI roughly corresponded to regions with vacuolar changes in brain tissue (Figure 1D,E).

**FIGURE 1:**
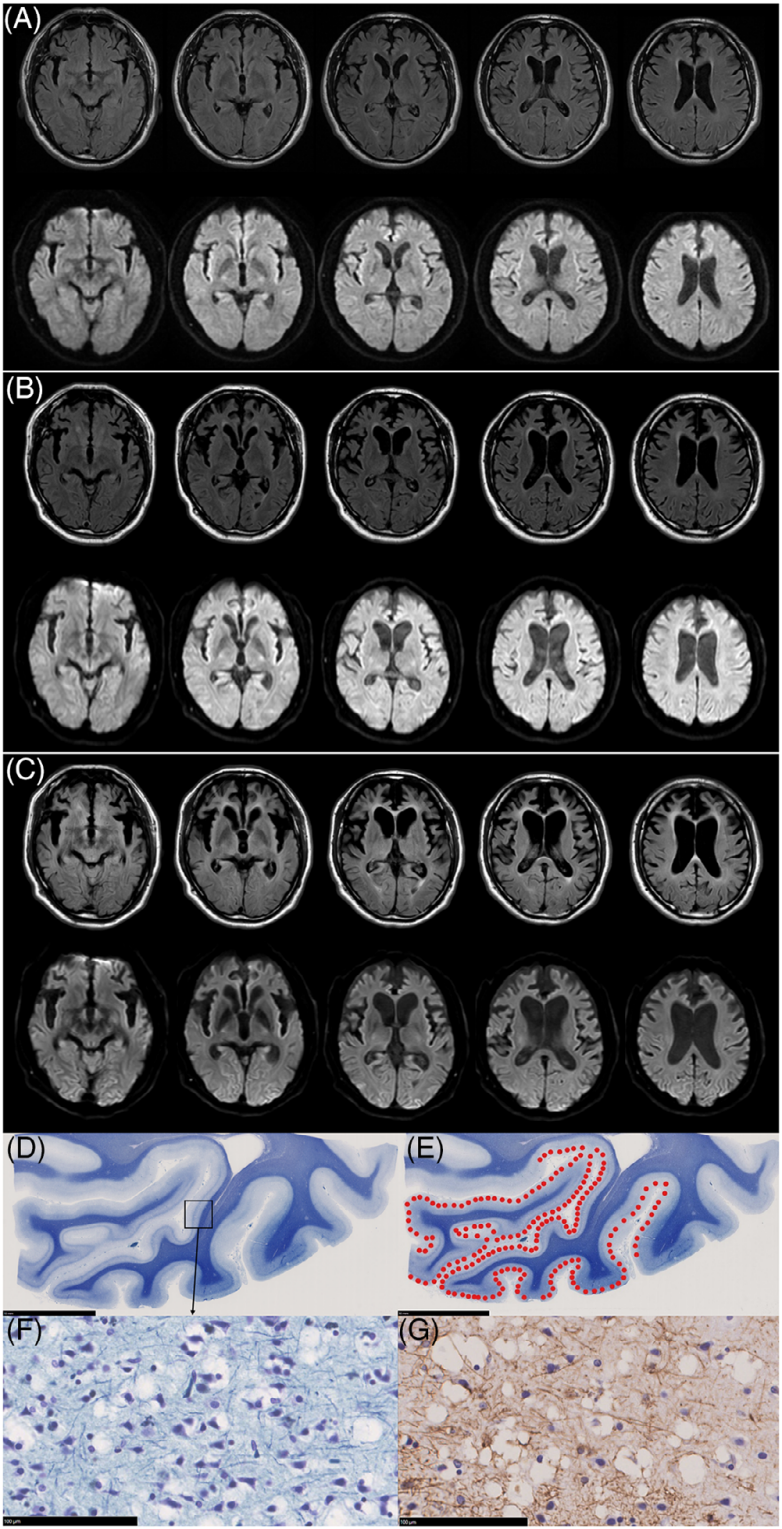
Longitudinal MRI findings and the distribution of vacuolar change in brain tissue. A, MRI findings at initial visit. No signal abnormalities in the occipital cortex on either FLAIR imaging (upper) or DWI (lower) were observed. B, MRI findings 3 years after the initial visit. Ribbon‐like hyperintensity in the occipital cortex on DWI (lower), with no apparent changes on FLAIR imaging (upper), was observed. C, MRI findings 10 years after the initial visit. Slight hyperintensity in the occipital cortex on FLAIR imaging (upper), in addition to persistent ribbon‐like hyperintensity on DWI (lower), was observed. D, The coronal section of the medial occipital cortex. Klüver–Barrea staining. Scale bars: 10 mm. E, The schematic distribution of vacuolar changes (red points), which are severe in the primary visual cortex and linsual gyrus. Klüver–Barrea staining. Scale bars: 10 mm. F, G, Vacuolar changes are seen in the second and third layers. Neuronal loss and astrogliosis are mild; (F) Klüver–Barrea staining, (G) glial fibrillary acidic protein immunostaining. Scale bars: 100 µm. DWI, diffusion‐weighted imaging; FLAIR, fluid‐attenuated inversion recovery; MRI, magnetic resonance imaging.

In Creutzfeldt–Jakob disease (CJD), hyperintensity on FLAIR imaging and DWI are characteristic imaging features,^4^ with DWI demonstrating higher diagnostic sensitivity.^5^ In early‐stage CJD, vacuolar change is the predominant neuropathological feature and is thought to underlie the high sensitivity of DWI.^5^ As the disease progresses, reactive astrogliosis becomes more prominent and may contribute to hyperintensity on FLAIR imaging.^5^ In our case, the MRI obtained after 3 years demonstrated DWI hyperintensity only, whereas the scan after 10 years showed hyperintensity on both DWI and FLAIR imaging. Histological examination revealed vacuolar changes that correlated with the DWI hyperintensity, as well as mild neuronal loss and reactive gliosis (Figure 1D‐G). These findings suggest that in VCP *D395G*‐associated vacuolar tauopathy, severe vacuolar change in the occipital cortex may initially present as DWI hyperintensity, followed later by FLAIR imaging hyperintensity as reactive astrogliosis develops. This temporal imaging evolution parallels the pattern observed in CJD.^6^ Moreover, neuronal loss and gliosis in the occipital cortex are generally mild in vacuolar tauopathy,^3^, ^7^ which may affect the conspicuity of FLAIR imaging hyperintensity. Of the five cases reported by Watanabe et al., only one showed hyperintensity on both FLAIR imaging and DWI; the other four showed only hyperintensity on DWI.^1^ As no autopsy was conducted in the case with both FLAIR imaging and DWI hyperintensity,^1^ the relationship between the neuropathological findings and MRI signals remains unclear. Thus, further research incorporating longitudinal MRI data and neuropathological examination is necessary to better understand the discrepancy between FLAIR imaging and DWI findings in vacuolar tauopathy.

Notably, in all cases reported by Watanabe et al., DWI signal changes were detected relatively early in the disease course.^1^ By contrast, in our case, MRI signal changes first appeared 3 years after the initial consultation and were also detected 10 years later (2 years before death), suggesting that signal changes may arise at various stages of the disease, either early or late. Further studies are required to determine whether this variability reflects differences in the timing of vacuolar change development in vacuolar tauopathy.

Our findings support the proposition that ribbon‐like hyperintensity on DWI, as proposed by Watanabe et al.^1^, is a characteristic feature of vacuolar tauopathy. However, our observations, along with previous reports,^1^ raise important concerns, such as the potential influence of scanner differences and imaging conditions. Further research, including longitudinal studies of VCP *D395G* mutation carriers using standardized imaging protocols, is necessary.

## AUTHOR CONTRIBUTIONS

Ryota Kobayashi analyzed radiological and neuropathological data and drafted the manuscript. Masafumi Kanoto analyzed radiological data and revised the manuscript. Shinobu Kawakatsu analyzed neuropathological data and revised the manuscript. Akihito Suzuki revised the manuscript. All authors have read and approved the final version of this manuscript.

## CONFLICT OF INTEREST STATEMENT

The authors have no conflicts of interest to report. Author disclosures are available in the supporting information.

## CONSENT TO PARTICIPATE

Written informed consent was obtained from the patient's wife for participation in this study and for publication of the data.

## ETHICS APPROVAL

This study was conducted in accordance with the principles of the Declaration of Helsinki and was approved by the Ethical Review Committee of Yamagata University Faculty of Medicine (approval number: 2021‐40).

## Supporting information



Supporting Information
